# Comprehensive analysis of BUBs gene family in lung adenocarcinoma with immunological analysis

**DOI:** 10.18632/aging.204517

**Published:** 2023-02-13

**Authors:** Xiaojuan Li, Tianqi Wang, Mi Li, Xing Bao, Tian Ma, Caihong Yang, Hua Wu, Hao Li

**Affiliations:** 1 Department of Orthopedics, Tongji Hospital, Tongji Medical College, Huazhong University of Science and Technology, Wuhan 430030, Hubei, China; 2 Department of Nephrology, Tongji Hospital Affiliated to Tongji Medical College, Huazhong University of Science and Technology, Wuhan 430030, Hubei, China; 3 Shenzhen Huazhong University of Science and Technology Research Institute, Shenzhen 518000, Guangdong, China

**Keywords:** BUBs gene family, lung adenocarcinoma, immunological analysis, survival analysis, pan-cancer

## Abstract

Lung adenocarcinoma (LUAD) is one of the most commonly malignant tumors, and major challenges remain in the treatment of LUAD. Budding uninhibited by benzimidazole 1/3 (BUB1/3) play significant roles in the process of spindle-assembly checkpoint (SAC) during mitosis. However, their roles in LUAD have not been established. Here, we performed an immunological analysis of BUB1/3 in LUAD using a comprehensive bioinformatics approach, quantitative real-time-PCR and Western blotting technique. Our results indicated that the expression levels of BUB1 and BUB3 in LUAD samples were higher than the expression levels in the control groups and were associated with some clinicopathologic parameters in patients with LUAD. BUB1/3 and their related genes were enriched in cell immune, and the immune infiltration analysis revealed that the BUB1/3 expression profile was significantly correlated with characteristics of immune cell infiltration. Survival analysis showed that the disease-free survival and overall survival of patients with LUAD decreased with an increase in the BUB1/3 expression levels. The mRNA and protein expression levels of BUB1 and BUB3 in each of the LUAD cell lines were upregulated to varying degrees. BUB1 and BUB3 are the potential immunological therapeutic intervention targets for patients with LUAD.

## INTRODUCTION

Lung cancer, as the second most common type of cancer, has become the main cause of cancer-related deaths in the world. Lung adenocarcinoma (LUAD) accounts for approximately 40% of lung cancer cases, making it the most common histological subtype of lung cancer [1]. The treatment strategies for LUAD depend on the understanding of the occurrence and progress of LUAD. The long-term prognosis of patients in their early stages of LUAD has been improved by surgery and neoadjuvant chemotherapy. However, a high rate of case fatality and a low five-year survival rate (<25%) has been observed for patients with LUAD [2, 3]. Targeted therapy is being increasingly used to treat patients with LUAD, and the use of this technique has significantly improved patient prognosis. Hence, new molecules should be identified and regulatory mechanisms associated with the occurrence and development of LUAD should be studied to identify new therapeutic targets.

The efficient and proper separation of chromosomes during cell division determines the genomic stability in all organisms. Chromosomes should be attached to microtubules emanating from the poles of the spindle for efficient separation at the onset of the anaphase stage of mitosis. The bio-orientation process of chromosomes is a random, error-prone process that often results in the generation of deviated intermediate kinetochores–microtubule interactions that must be detected and corrected [4]. Thus, the spindle assembly checkpoint (SAC) mechanism exists to monitor this process. SAC is mediated by a group of highly conserved signal transduction proteins, such as Budding Uninhibited by Benzimidazoles (BUBs), mitotic arrest deficient1/2/3 (MAD1/2/3) and monopolar spindle1 (MPS1). These proteins are situated on the kinetochore of the chromosomes (that have not yet to formed bipolar attachments) and generate checkpoint signals.

BUB1 and BUB3, belonging to the BUBs gene family, dictate the functions of the spindle assembly checkpoint associated with mitosis. The process of chromosome aggregation is controlled by BUB1 which also regulates the centromeric localization process and is highly significant in the establishment and maintenance of the efficient bipolar attachment process (to realize the attachment to spindle microtubules) [5]. By binding to BUB1 and forming a BUB1/BUB3 complex, BUB3 plays a role in the inhibition of the ubiquitin ligase of anaphase promoting complex C (APC/C) which later promotes the functioning and formation of complex C [6, 7]. The spatial separation of kinase and phosphatase activities within the BUB complex is necessary to balance its functions as a checkpoint and in chromosome alignment [8]. Dysregulation of BUB1/3, the key mediators of spindle assembly checkpoints, has been reported to result in the incidence and development of various types of cancers, such as stomach cancer, leukemia, liver cancer, and breast cancer [9, 10]. However, the role of BUB1/3 in lung adenocarcinoma remains unclear.

In this study, we addressed this issue by identifying the transcriptional and protein expression patterns of BUB1/3 based on the Oncomine database, The Cancer Genome Atlas (TCGA) database, the Genotype-Tissue Expression (GTEx) database and the human protein atlas (HPA). We further evaluated Gene Ontology (GO) functions and Kyoto Encyclopedia of Genes and Genomes (KEGG) pathways of BUB1/3 and the associated differential expression genes (DEGs) in LUAD. Furthermore, we analyzed the immune infiltration, protein-protein interaction (PPI) network analysis, clinicopathology, and prognostic value of BUB1/3 in LUAD. We also conducted *in vitro* experimental verification of LUAD cell lines to evaluate the expression levels of BUB1/3 in LUAD cell lines. Therefore, our study clarifies the biological functionality, immunological analysis, and prognostic value of BUB1/3 to improve the diagnosis and treatment of LUAD.

## RESULTS

### Expression profile of BUB1/3 in pan-cancer tissues

**Figure 1:**
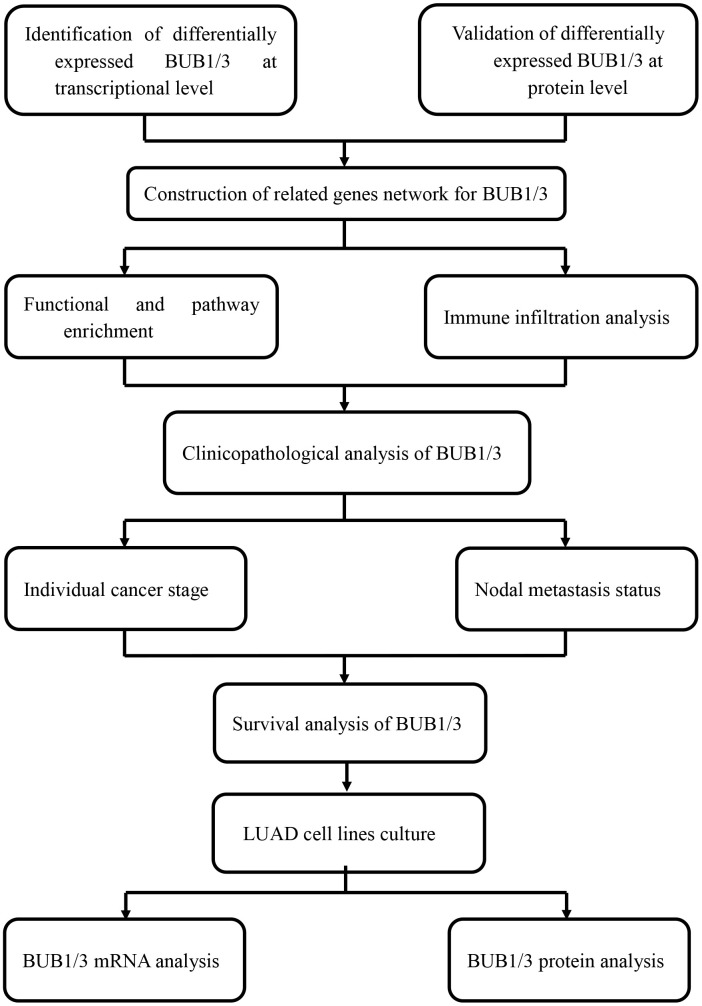
Flow chart presenting the analytical process.

**Figure 2:**
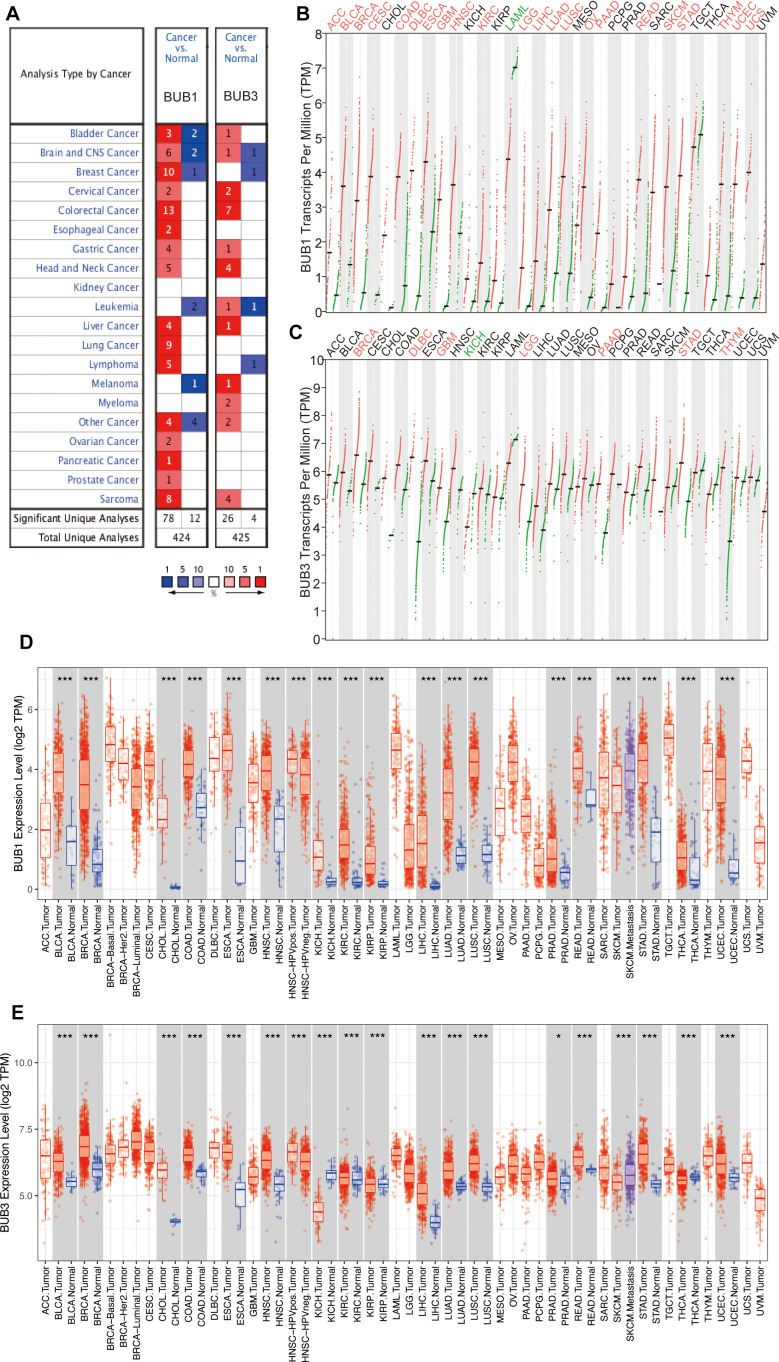
**Differential expression of BUB1/3 at transcriptional levels in different cancers.** (**A**) BUB1/3 mRNA expression in 20 different types of cancers analyzed using the data in the Oncomine platform. Red represents high expression levels, and blue represents low expression levels. *p* <0.0001, multiple change = 2, gene grade = 10%. (**B**, **C**) Expression levels of BUB1 (**B**) and BUB3 (**C**) mRNAs in 33 cancer tissues and paired normal tissues obtained from the GEPIA database. *p* <0.05, | Log2FC | > = 2. (**D**, **E**) Expressions levels of BUB1 (**D**) and BUB3 (**E**) in cancer tissues and the corresponding normal tissues obtained from the TIMER database. Red, cancerous tissue; Blue, normal tissue. *, *p* <0.05; **, *p* <0.01; ***, *p* <0.001.

Figure 1 presents a flow chart of the process followed to conduct the studies. We analyzed data from the Oncomine, Gene Expression Profiling Interactive Analysis (GEPIA), and Tumor Immune Estimation Resource (TIMER) databases to study the expression profiles of BUB1/3 in different cancer tissues. The expression profiles in the corresponding normal tissues were also studied for comparison. Analysis of the data obtained from the Oncomine database revealed that the level of BUB1 expression was significantly high in the central nervous system, bladder, cervical, brain, ovarian, breast, gastric, colorectal, esophageal, head, pancreatic, liver, neck, lung, and prostate, cancers, as well as lymphomas, and sarcoma cancers. Furthermore, the expression level of BUB1 was higher than that in normal tissues (Figure 2A). Additionally, the expression level of BUB3 was significantly higher in cases of cervical, bladder, head, brain, colorectal, central nervous system, gastric, head, blood, and liver cancers, as well as melanomas and sarcomas. However, the expression level of BUB3 in lung cancer did not differ significantly from that recorded in normal lung tissues (Figure 2A). The data of 33 types of cancers were obtained from the TIMER and GEPIA databases and further analyzed to verify these results above. Our findings were consistent with those of previous reports (Figure 2B–2E). Overall, these results suggest that BUB1 is highly expressed in most cancer cells, including LUAD. These findings provide a platform for conducting further studies on the BUBs gene family as a prognostic factor of LUAD.

### High expression of BUB1/3 mRNA in LUAD

**Figure 3:**
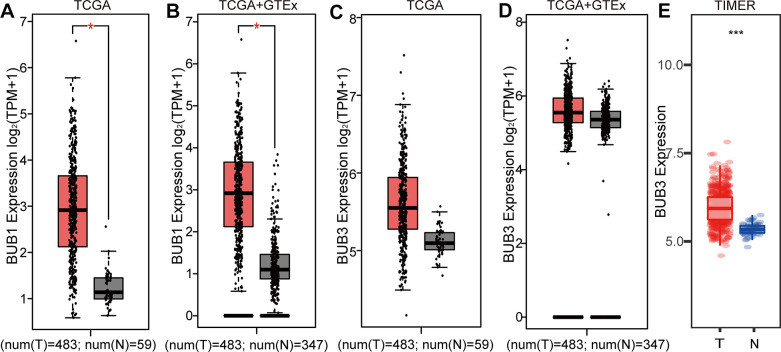
**Differential expression of BUB1/3 at the transcriptional level in LUAD.** (**A**, **B**) LUAD mRNA expression obtained by analyzing the GEPIA database and BUB1 mRNA expression in paired normal tissues. *p* <0.05, | Log2FC | > = 2. (**C**, **D**) LUAD mRNA expression in GEPIA and BUB3 mRNA expression in paired normal tissues. *p* values <0.05, | Log2FC | > = 2. (**E**) Expression of LUAD and BUB3 mRNA in paired normal tissues (data obtained from the TIMER database). *p* value <0.0001.

The BUB1/3 mRNA expression levels in LUAD were further analyzed using data from various databases. As shown in Figure 3A, the BUB1 mRNA expression was upregulated in LUAD compared with normal tissues from TCGA database. This result was consistent with the previous result, based on the TCGA and GTEx databases (Figure 3B). Five studies based on the Oncomine database revealed that the BUB1 mRNA expression in the LUAD tissues was higher than that in the normal tissues. The multiple changes were 2.851, 4.125, 2.676, 2.707, and 2.005 (*p* = 7.58E-8, 8.15E-14, 1.83E-6, 4.07E-14, and 3.27E-13, respectively) (Table 1). No statistically significant differences were observed in the BUB3 mRNA expression levels in the case of LUAD. These results were compared with the expression levels recorded for the normal tissues in the TCGA and integrated TCGA and GTEx databases (Figure 3C, 3D). However, when the differences in the BUB3 expression levels between the LUAD and normal tissues were compared using the TIMER database, a statistically significant difference in the mRNA expression of BUB3 was observed (Figure 3E). In conclusion, BUB1 and BUB3 mRNAs were found to be highly expressed in LUAD.

**Table 1:** Five Oncomine studies revealing BUB1 mRNA expression profiles.

	**Types of LUAD VS. lung**	**Fold change**	**P-value**	**T-test**	**Ref**
BUB1	Lung adenocarcinoma	2.851	7.58E-8	7.238	Garber
	Lung adenocarcinoma	4.125	8.15E-14	9.473	Hou
	Lung adenocarcinoma	2.676	1.83E-6	5.254	Su
	Lung adenocarcinoma	2.707	4.07E-14	11.122	Okayama
	Lung adenocarcinoma	2.005	3.27E-13	8.748	Landi

### High protein expression of BUB1/3 associated with LUAD

**Figure 4:**
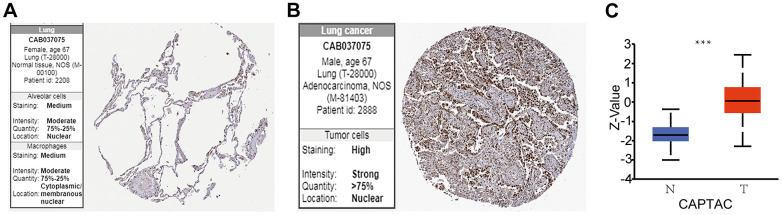
**BUB3 protein expression levels in LUAD.** (**A**, **B**) Protein expression of BUB3 in tumor and normal tissues; data obtained from the HPA. (**C**) Protein expression of BUB3 in LUAD and normal tissues; data obtained from the CAPTAC database (* *p* <0.05). The Z-value represents the standard deviation from the median (for the cancer types under being studied). The Log2 spectral count ratio from CAPTAC was first normalized for each sample profile. It was then normalized among the samples.

After analyzing the BUB1/3 mRNA expression profiles, we studied the BUB1/3 protein expression profile using the HPA and Clinical Proteomic Tumor Analysis Consortium (CPTAC) databases. However, the BUB1 protein expression in LUAD was not present in either database. Meanwhile, BUB3 protein was expressed in alveolar nuclei, cytoplasm, and nuclear membranes of macrophages (associated with normal lung tissue) (Figure 4A). The expression of the BUB3 protein was found to be significantly upregulated in the nuclear membrane of the tumor cells associated with LUAD (Figure 4B). We obtained similar results using the CPTAC database (Figure 4C). In summary, these results revealed that the protein level expression of BUB3 in LUAD was significantly higher than that of the normal tissues.

### Functional enrichment of BUB1/3 in LUAD

**Figure 5:**
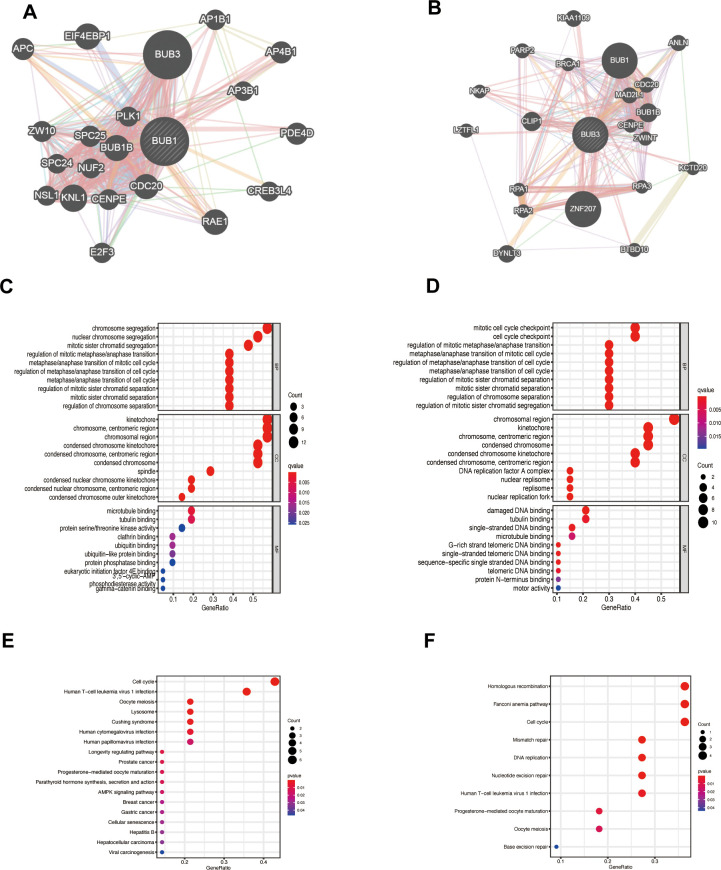
**Function of the genes significantly associated with BUB1/3.** (**A**, **B**) PPI networks constructed for BUB1 (**A**), BUB3 (**B**), and the 20 related genes using the data presented in the GeneMANIA database. (**C**, **D**) Prediction of the function of the related genes in the BUB1 (**C**) and BUB3 (**D**) PPI network through GO analyses. (**E**, **F**) Function of the related genes in the BUB1 (**E**) and BUB3 (**F**) PPI network predicted through KEGG analyses.

The GeneMANIA platform was used to construct gene networks of BUB1/3 and their 20 related gene networks (Figure 5A, 5B). The WebGestalt database was used to analyze the GO and KEGG functions of BUB1/3 and the 20 related genes. Some functions were enriched in human T-cell leukemia virus type 1 infection, human cytomegalovirus infection, viral carcinogenesis, and AMPK signaling pathways (Figure 5C, 5D). This finding provides a framework to study the relationship between the extent of immune infiltration achieved and the BUB gene family.

### Relationship between the level of BUB1/3 expression and the level of immune infiltration achieved in LUAD

**Figure 6:**
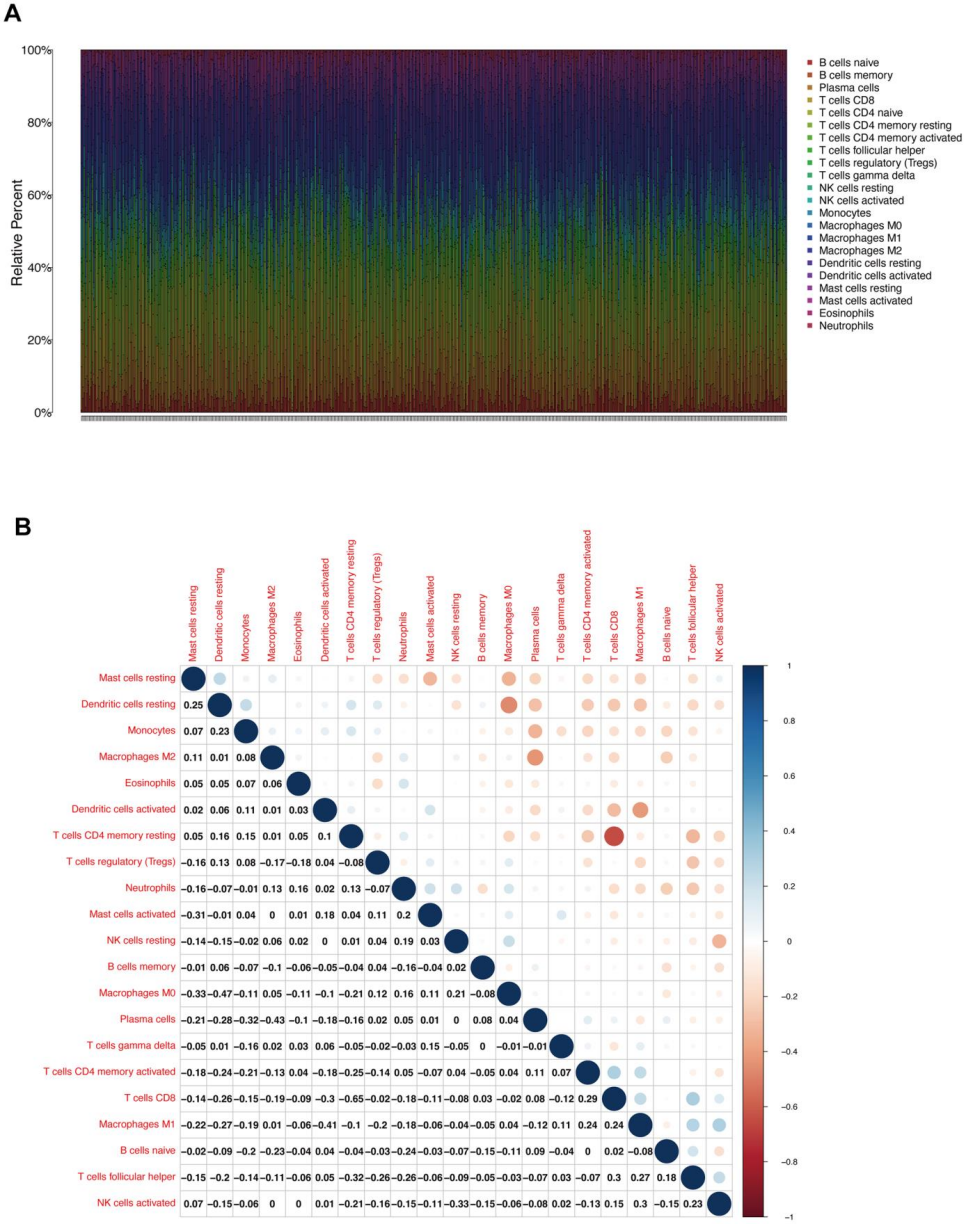
**TIC profile and correlation analysis.** (**A**) Bar chart showing the proportion of the 22 types of TIC in patients with LUAD (**B**) Heat map showing the correlation between two types of TIC cells corresponding to the 22 species under study and the related *p* values.

**Figure 7:**
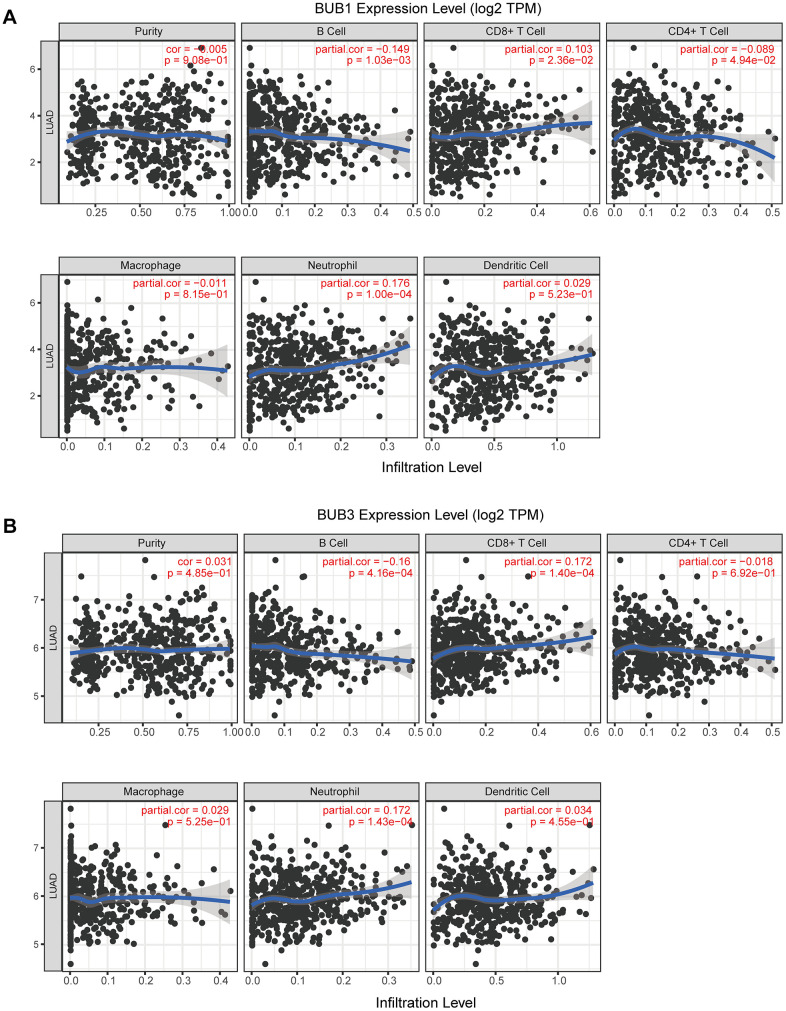
**Correlation analysis of BUB1/3 and extent of immune cell infiltration.** (**A**, **B**) Correlation analysis for BUB1 (**A**) and BUB3 (**B**), taking into account tumor purity, B cells, CD8+ T cells, CD4+ T cells, macrophages, neutrophils, and dendritic cells.

Next, we used TIMER database to determine whether BUB1/3 mRNA expression was related to immune cell infiltration. First, an atlas consisting of 22 types of immune cells was constructed (Figure 6A). The heat map in Figure 6B showed the relationships of the 22 immune cells that infiltrate the tumor cells in each capsule. A ***p***-value was obtained to describe the relationship between each set of two immune cells was obtained. Figure 7 shows that BUB1/3 mRNA expression was not related to tumor purity. However, BUB1 mRNA expression was significantly correlated with B cells, neutrophils, CD4+T cells, and CD8+T cells (Figure 7A) BUB3 mRNA expression was significantly correlated with CD8+ T cells, B cells, and neutrophils (Figure 7B).

### Relationship between BUB1/3 expression and the clinicopathological features of LUAD

**Figure 8:**
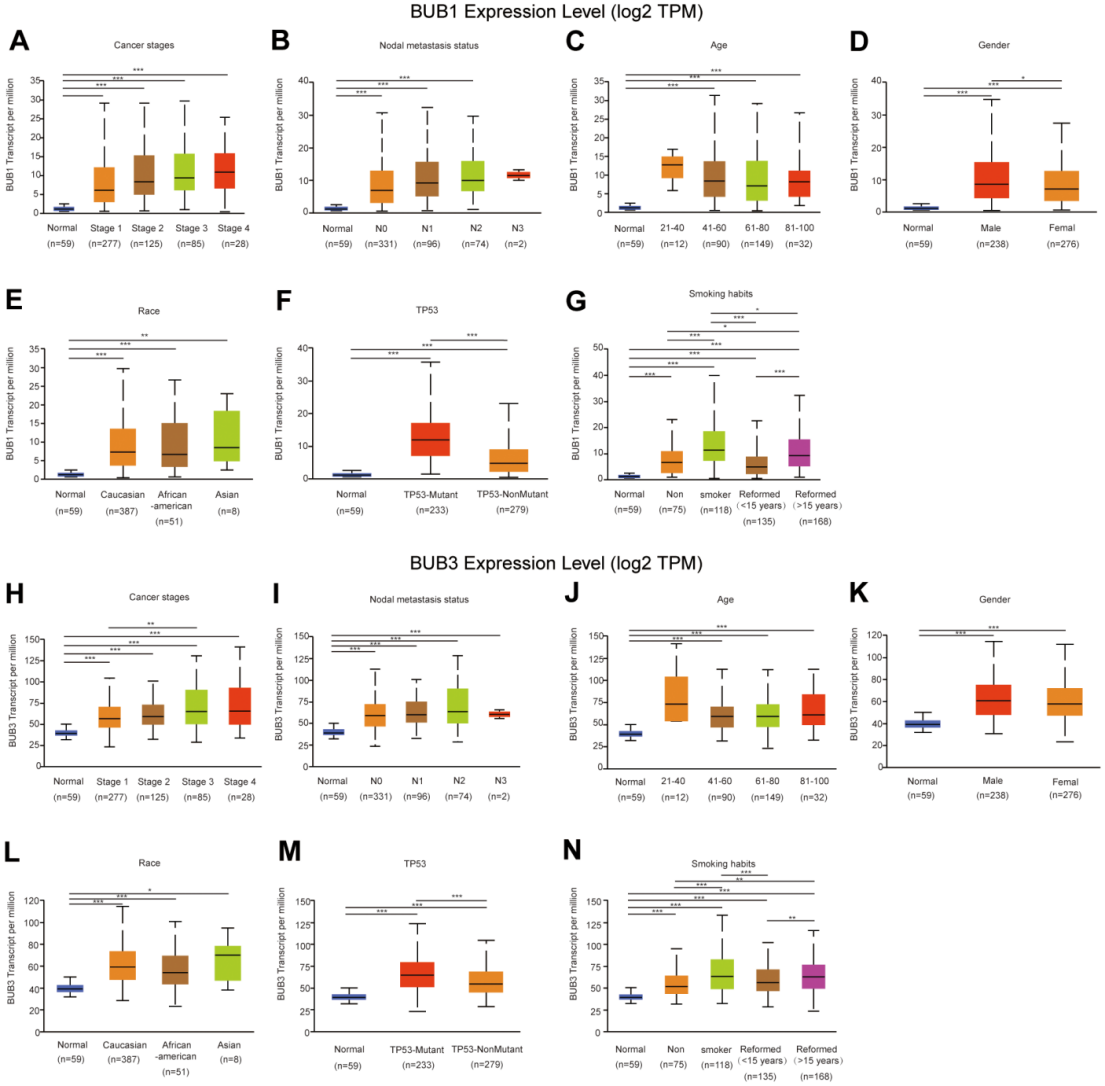
**Relationship between the level of BUB1/3 expression and the clinicopathological parameters of the patients with LUAD.** We analyzed the relationship between BUB1 (**A**–**G**) and BUB3 (**H**–**N**) expression levels and the cancer stage, lymph node metastasis status, age, sex, race, TP53, and smoking habit of the subjects.

The UALCAN database was analyzed to determine the relationship between the level of BUB1/3 expression and the clinicopathological parameters of the patients with LUAD. The parameters included cancer stage, lymph node metastasis status, age, sex, race, TP53 mutation status, and smoking habit (Figure 8 and Table 2). BUB1 expression level in stages 1, 2, 3, and 4 tissues was significantly higher than that in normal tissues. No significant difference was observed in the BUB1 expression levels among different stages (Figure 8A). Figure 8B revealed that BUB1 expression was significant correlated significantly with the lymph node metastasis status. The expression levels of BUB1 in N0, N1, and N2 cancers was higher than that in the normal tissues. However, a significant difference in between the expression levels of BUB1 in N3 and normal tissues was not observed, which could be attributed to the small sample size of N3 tissues. Furthermore, no significant difference was observed in the BUB1 expression levels according to lymph node metastasis statuses. We further analyzed the relationships between the BUB1 expression levels and different subgroups according to age, sex, race, TP53 mutation, and smoking habit. The results of the subgroup analysis by age revealed that high BUB1 expression was statistically significant among patients > 41 years of age (Figure 8C). Moreover, BUB1 expression was significantly higher in male patients than that in female patients (Figure 8D). The expression level of BUB1 in different ethnic subgroups was higher than that in the normal group. However, no statistical difference was observed among the different ethnic subgroups (Figure 8E). The expression level of BUB1 in the TP53 mutant and non-mutant subgroups was significantly higher than that of the normal group, and the expression of BUB1 in the TP53 mutant subgroup was significantly higher than that in the non-mutant subgroup (Figure 8F). Similarly, BUB1 expression level in patients with and without smoking habits was higher than that of the normal group. BUB1 expression was also higher in the smoking subgroup than that in the non-smoking subgroup. Finally, BUB1 expression level in amended smokers was significantly lower than that in smokers (Figure 8G).

**Table 2:** Clinicopathological factors associated with BUB1 in LUAD.

	**Levels**	**Low expression of BUB1**	**High expression of BUB1**	**p**
n		267	268	
T stage, n (%)	T1	105 (19.7%)	70 (13.2%)	0.003
	T2	123 (23.1%)	166 (31.2%)	
	T3	27 (5.1%)	22 (4.1%)	
	T4	10 (1.9%)	9 (1.7%)	
N stage, n (%)	N0	188 (36.2%)	160 (30.8%)	0.005
	N1	40 (7.7%)	55 (10.6%)	
	N2	26 (5%)	48 (9.2%)	
	N3	0 (0%)	2 (0.4%)	
M stage, n (%)	M0	179 (46.4%)	182 (47.2%)	0.023
	M1	6 (1.6%)	19 (4.9%)	
Pathologic stage, n (%)	Stage I	162 (30.7%)	132 (25%)	0.007
	Stage II	58 (11%)	65 (12.3%)	
	Stage III	34 (6.5%)	50 (9.5%)	
	Stage IV	7 (1.3%)	19 (3.6%)	
Primary therapy outcome, n (%)	PD	22 (4.9%)	49 (11%)	0.004
	SD	22 (4.9%)	15 (3.4%)	
	PR	4 (0.9%)	2 (0.4%)	
	CR	176 (39.5%)	156 (35%)	
Gender, n (%)	Female	159 (29.7%)	127 (23.7%)	0.006
	Male	108 (20.2%)	141 (26.4%)	
Race, n (%)	Asian	3 (0.6%)	4 (0.9%)	0.864
	Black or African American	28 (6%)	27 (5.8%)	
	White	213 (45.5%)	193 (41.2%)	
Age, n (%)	<=65	119 (23.1%)	136 (26.4%)	0.113
	>65	141 (27.3%)	120 (23.3%)	
number_pack_years_ smoked, n (%)	<40	101 (27.4%)	87 (23.6%)	0.004
	>=40	69 (18.7%)	112 (30.4%)	
Smoker, n (%)	No	49 (9.4%)	26 (5%)	0.005
	Yes	210 (40.3%)	236 (45.3%)	
OS event, n (%)	Alive	186 (34.8%)	157 (29.3%)	0.010
	Dead	81 (15.1%)	111 (20.7%)	
DSS event, n (%)	Alive	203 (40.7%)	176 (35.3%)	0.008
	Dead	47 (9.4%)	73 (14.6%)	
PFI event, n (%)	Alive	172 (32.1%)	137 (25.6%)	0.002
	Dead	95 (17.8%)	131 (24.5%)	

We also investigated the BUB3 expression levels in similar subgroups (Figure 8 and Table 3). BUB3 expression levels recorded for the stage 1, 2, 3, and 4 tissues were significantly higher than that in normal tissues, and a significant difference was observed between the results of stage 1 and stage 3 (Figure 8H). As shown in Figure 8I, BUB3 expression level was correlated with the status of lymph node metastasis. BUB3 expression level was higher in N0, N1, N2, and N3 cancers than that in normal tissues. Regarding age, the expression level of BUB3 in the subgroup of patients >41 years of age was statistically significant (Figure 8J). BUB3 expression levels in both male and female patients with LUAD were higher than those in normal subjects, and no significant difference was observed in the BUB3 expression levels between male and female patients (Figure 8K). Moreover, the expression of BUB3 among the different ethnic subgroups was higher than that of the normal group. However, no statistical difference was observed among the different ethnic subgroups (Figure 8L). BUB3 expression in the TP53 mutant and non-mutant subgroups was significantly higher than that of the normal group. Additionally, BUB3 expression was significantly higher in the TP53 mutant subgroup than the non-mutant subgroup (Figure 8M). BUB3 expression level in the smoking and non-smoking patients with LUAD was significantly higher than that of the normal group, and BUB3 expression level of the smoking subgroup was higher than that of the non-smoking subgroup (Figure 8N). In conclusion, BUB1/3 were associated with various clinicopathological characteristics of patients with LUAD. These results may help develop a method that can be used for the clinical prognosis of the patients with LUAD.

**Table 3:** Clinicopathological factors associated with BUB3 in LUAD.

**Characteristic**	**Levels**	**Low expression of BUB3**	**High expression of BUB3**	**p**
n		267	268	
T stage, n (%)	T1	108 (20.3%)	67 (12.6%)	< 0.001
	T2	129 (24.2%)	160 (30.1%)	
	T3	23 (4.3%)	26 (4.9%)	
	T4	5 (0.9%)	14 (2.6%)	
N stage, n (%)	N0	181 (34.9%)	167 (32.2%)	0.223
	N1	44 (8.5%)	51 (9.8%)	
	N2	29 (5.6%)	45 (8.7%)	
	N3	1 (0.2%)	1 (0.2%)	
M stage, n (%)	M0	177 (45.9%)	184 (47.7%)	0.149
	M1	8 (2.1%)	17 (4.4%)	
Pathologic stage, n (%)	Stage I	159 (30.2%)	135 (25.6%)	0.016
	Stage II	64 (12.1%)	59 (11.2%)	
	Stage III	31 (5.9%)	53 (10.1%)	
	Stage IV	9 (1.7%)	17 (3.2%)	
Primary therapy outcome, n (%)	PD	26 (5.8%)	45 (10.1%)	0.053
	SD	21 (4.7%)	16 (3.6%)	
	PR	3 (0.7%)	3 (0.7%)	
	CR	180 (40.4%)	152 (34.1%)	
Gender, n (%)	Female	151 (28.2%)	135 (25.2%)	0.178
	Male	116 (21.7%)	133 (24.9%)	
Race, n (%)	Asian	3 (0.6%)	4 (0.9%)	0.693
	Black or African American	31 (6.6%)	24 (5.1%)	
	White	208 (44.4%)	198 (42.3%)	
Age, n (%)	<=65	129 (25%)	126 (24.4%)	0.998
	>65	131 (25.4%)	130 (25.2%)	
number_pack_years_smoked, n (%)	<40	82 (22.2%)	106 (28.7%)	0.239
	>=40	91 (24.7%)	90 (24.4%)	
Smoker, n (%)	No	47 (9%)	28 (5.4%)	0.024
	Yes	213 (40.9%)	233 (44.7%)	
OS event, n (%)	Alive	190 (35.5%)	153 (28.6%)	< 0.001
	Dead	77 (14.4%)	115 (21.5%)	
DSS event, n (%)	Alive	207 (41.5%)	172 (34.5%)	0.004
	Dead	47 (9.4%)	73 (14.6%)	
PFI event, n (%)	Alive	169 (31.6%)	140 (26.2%)	0.012
	Dead	98 (18.3%)	128 (23.9%)	

### Receiver operating characteristic (ROC) analysis and prognostic value of BUB1/3

**Figure 9:**
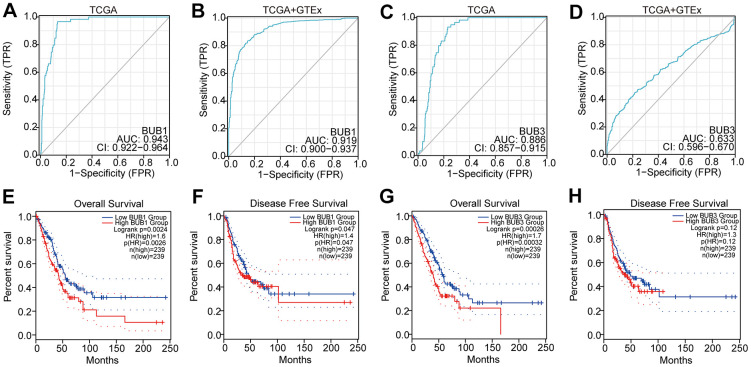
**ROC analysis and prognostic effect of BUB1/3 for LUAD.** (**A**, **B**) The diagnostic efficacy of BUB1 for LUAD using the TCGA database and the TCGA database combined with the GTEx database. (**C**, **D**) The diagnostic efficacy of BUB3 for LUAD both the TCGA database and the TCGA combined with the GTEX database. (**E**, **F**) Relationship between BUB1 expression level and overall survival (**A**) and disease-free survival (**B**) in patients with LUAD (**G**, **H**) Relationship between BUB3 expression level and overall survival (**C**) and disease-free survival (**D**) in patients with LUAD.

Through ROC analysis, we determined the diagnostic efficacy of BUB1/3 for LUAD. We found that BUB1 expression could serve as a potential predictor with high accuracy for LUAD in both the TCGA database (area under the curve [AUC] =0.943, confidence interval [CI]: 0.922-0.964) and the TCGA combined with the GTEx database (AUC = 0.919, CI: 0.900-0.937) (Figure 9A, 9B). BUB3 expression exhibited potential as a predictor with certain accuracy for LUAD in the TCGA database (AUC=0.886, CI: 0.857-0.915), and low accuracy for LUAD in the TCGA combined with the GTEx database (AUC = 0.633, CI: 0.596-0.670) (Figure 9C, 9D).

The Kaplan–Meier plotter was used to analyze the relationship between BUB1/3 expression level and the prognosis of patient with LUAD s. As shown in Figure 9A, an increase in the expression of BUB1 (HR [high] = 1.6, log-rank *p* = 0.0024) resulted in a decrease in the overall survival (OS) of the patients with LUAD (Figure 9E). A high BUB1 expression level (HR [high] = 1.4, and Llog-rank *p* = 0.047) was also associated with a short disease free survival (DFS) (Figure 9F). Figure 9G showed that high BUB3 expression (HR [high] = 1.7, and log-rank *p* = 0.00026) was associated with a short OS in patients with LUAD (Figure 9G). However, a high BUB3 expression level was not associated with a short DFS in patients with LUAD (Figure 9H). These results suggest that the BUB1/3 expression level significantly influences the prognosis of patients with LUAD. Therefore, the expression of BUB1/3 may serve as a useful biomarker for predicting the survival of patients with LUAD.

### Expression of BUB1/3 (*in vitro*) in LUAD cell lines

**Figure 10:**
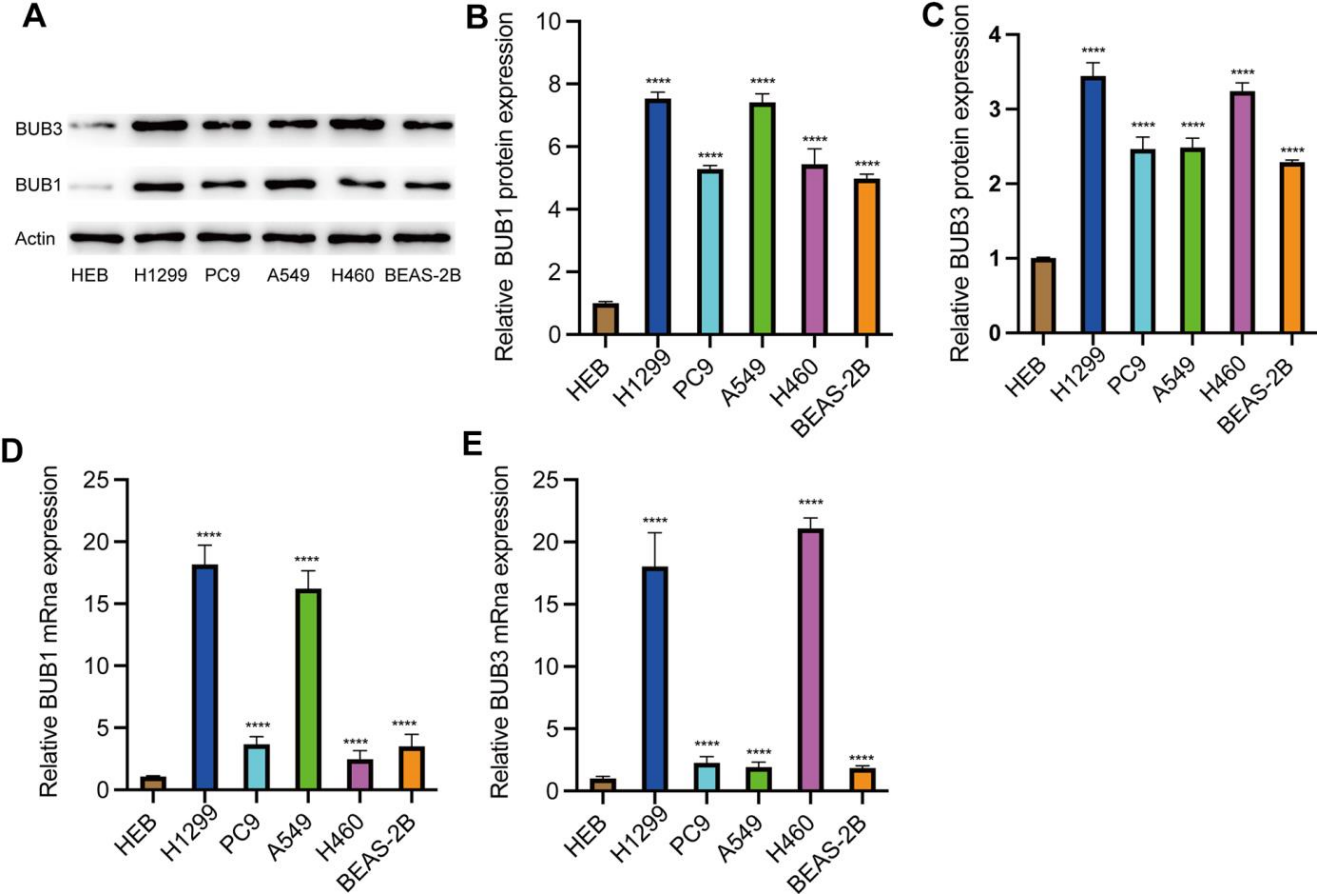
**Expression of BUB1/3 in LUAD cell lines.** (**A**–**C**) The protein expression levels of BUB1/3 in the normal HBE cell line, human LUAD cell line. ****, *p* <0.0001. (**D**, **E**) The mRNA expression levels of BUB1/3 in the normal HBE cell line, human LUAD cell line. ****, *p* <0.0001.

We also evaluated the BUB1/3 expression levels by conducting *in vitro* studies with LUAD cell lines. Therefore, the mRNA and protein expression levels of BUB1/3 in the normal human bronchial epithelial (HBE) cell line and human LUAD cell lines A549, H1299, PC9, HCC827, and BEAS-2B were analyzed. Figure 10A–10C shows the protein expression levels of BUB1 and BUB3 in HBE, H1299, PC9, A549, H460, and BEAS-2B cell lines. The results revealed that the BUB1 and BUB3 expression levels in the LUAD cell lines were upregulated to varying degrees compared to the expression levels observed in normal bronchial epithelial cells. The qPCR technique was used to detect the mRNA expression levels of BUB1/3 in each cell line. The results were consistent with those of previous reports. Specifically, the mRNA expression levels of BUB1 and BUB3 in each of the LUAD cell lines were upregulated to varying degrees (compared to the expression levels observed in the normal bronchial epithelial cells) (Figure 10D, 10E). We also verified the high expression levels of BUB1/3 in LUAD through *in vitro* cell experiments.

## DISCUSSION

The process of mitosis is significantly affected by the BUB1, and chromosomal arm separation and chromosomal passenger complex localization are catalyzed by BUB1. Deviation from its normal expression level can result in errors in chromosome segregation, aneuploidy, and cancer susceptibility. Disturbances in the BUB gene family have been reported in many types of cancers. While, abnormal expression of BUB1/3 has been found in many studies, the exact mechanism remains unclear. High expression of BUB1 and BUB3 in low-grade breast cancers was associated with longer overall survival [11, 12]. Additionally, BUB1/3 are simultaneously overexpressed in gastric tumors and exhibit a significant correlation with Ki-67 expression [13]. BUB1 was found to modulate the G2/M transition to promote the proliferation of non-muscle-invasive bladder cancer cells [14] and drove the progression and proliferation of bladder cancer by regulating the transcriptional activation of STAT3 signaling [15]. Moreover, BUB1 promotes the proliferation and vertical migration ability of liver cancer cells by activating phosphorylation of SMAD2 [16, 17]. DUXAP8 serves as a sponge of MiR-490-5p to promote the expression of BUB1 in hepatocellular carcinoma [18]. Inhibition of BUB1 suppresses cell proliferation, and tumor growth, cell migration, and invasion and induces apoptosis of osteosarcoma cells by blocking the PI3K/AKT and ERK signalling pathways [19]. BUB1 overexpression promotes the clonogenic potency of human myeloma-derived cell lines [20]. BUB1 overexpression weakens KIF4A knockdown-mediated effects on cell viability, colony formation, migration, and apoptosis in ovarian cancer [21]. FDI-6 reverses olaparib-induced adaptive resistance and inhibits cell cycle progression and DNA damage repair by repressing the expression of BUB1 in pancreatic cancer [22]. In our study, the expression of BUB1 mRNA was significantly elevated in the central nervous system, bladder, cervical, brain, ovarian, breast, gastric, colorectal, esophageal, head, pancreatic, liver, neck, lung, and prostate cancers, as well as lymphomas and sarcomas. The expression level of BUB3 was significantly elevated in cases of cervical, bladder, head, brain, colorectal, central nervous system, gastric, head, blood, and liver cancer.

LUAD is one of the most common types of malignant tumors worldwide, and the incidence of LUAD increases each year. Patients with LUAD cannot be effectively treated by traditional treatment methods, i.e., surgery, radiation, and chemotherapy, due to the high rates of recurrence and metastasis among patients with LUAD. Although immunotherapy can be used to improve the survival rate of patients with LUAD, the five-year overall survival rate is as low as 23%. Therefore, the pathogenesis of LUAD should be further elucidated to find new and effective treatment strategies.

The effect of BUB1/3 on the incidence and development of LUAD is not well understood. BUB1B, in conjunction with ZNF143, regulates glycolysis in LUAD, and overexpression of BUB1B had increases the proliferation, migration, and invasion of LUAD cells [23]. To the best of our knowledge, this is the first study to explore the patterns of expression and prognostic value of BUB1/3 in LUAD. Several researchers have reported that the low expression levels of these genes help delay tumor growth and improve the survival rate of patients with LUAD. The results of the analyses of the Oncomine, GEPIA, and TIMER datasets revealed that the expression of BUB1 mRNA in LUAD was higher than that in normal tissues. Analysis of the HPA and the CPTAC databases revealed high protein expression levels of BUB3 in LUAD. Therefore, we inferred that the disease-free survival and overall survival rates of the patients with LUAD would decrease with an increase in the BUB1 expression levels.

The BUB3 gene can function in tandem with the BUB1 gene. The BUB3 gene becomes functional when it binds to the BUB1 gene. A positive correlation was found between BUB3 and BUB1 gene expression, suggesting that the BUBs genes act as a complex during the process of mitosis. Moreover, the function of anaphase promoting complex (APC) is inhibited by the BUB1/BUB3 complex. Under these conditions, the entry into the anaphase phase of the cycle and the exit from the mitosis are inhibited. These processes are crucial for the realization of efficient chromosome separation. The BUB3 gene has been found to be overexpressed in cases of gastric cancer, oral carcinoma, lung cancer, and prostate cancer. Overexpression of BUB3 was detected in oral squamous cell carcinoma and was associated with increased cellular proliferation [24]. The positive expression of cytoplasmic BUB3 protein was significantly related to the recurrence of prostate carcinomas [25]. Moreover, the mutation or haploinsufficiency of BUB1 or BUB3 led to an increased risk of colorectal cancer at a young age [26]. DMAP1 was highly phosphorylated in pancreatic cancer cells, which impeded DMAP1/BUB3 interaction and the relevant cellular activity [27].

The role of BUB3 in the incidence and development of lung cancer has yet to be elucidated. MST1/2 via BUB3 affects the remodeling of pulmonary arterial adventitial [28]. BUB3 directly binds to LNC CRYBG3 and interrupts its interaction with CDC20 to result in aneuploidy, thus promoting the tumorigenesis and metastasis of lung cancer cells [29]. The expression of BUB3 mRNA was found to be higher in lung tumor tissues than non-malignant lung tissues [30]. The expression level of Bub3 is highly related to the incidence of non-small cell lung cancer [29]. In the current study, we reveal a positive association between the BUB3 gene and human LUAD and the lung adenocarcinoma cell lines. We also reported that a high level of BUB3 expression is correlated with a decrease in the OS and DFS. The protein and mRNA expression of BUB1/3 were found to be upregulated in different LUAD cell lines, however, the specific mechanism requires further investigation. The results reported herein indicate that the BUB3 and BUB1 genes can potentially function as oncogenes and carry prognostic values. Therefore, LUAD may be properly diagnosed, and the chances of survival of the patients with LUAD may be improved by studying these genes. Because high expression of BUB1/3 can potentially indicate a high degree of malignancy in patient with LUAD, these patients should be subjected to radical treatment methods.

This study had several limitations. First, the analysis was conducted using LUAD cell line samples (i.e., tumor samples from patients were not collected from patients). Therefore, whether the observations of BUB can be detected in tumor samples from patients with LUAD remains unclear. Future studies should be conducted with a focus on the levels of expression of the BUBs in tumor samples. This could potentially help develop a convenient screening method for the diagnosis of LUAD. Until then, targeted gene therapy and the genes belonging to the BUB family can be exploited to treat LUAD.

## MATERIALS AND METHODS

### Expression of the BUB gene family at the transcriptional level between different tumor samples and the corresponding normal samples

The Oncomine (https://www.oncomine.org/) database was analyzed to determine the gene expression characteristics of BUB1/3 mRNA expression profiles in 20 cancer samples and the corresponding normal samples [31]. The data were compared using a t-test. An in-depth analysis of the TCGA database (https://www.cancer.gov/about-nci/organization/ccg/research/structural-genomics/tcga) and GTEx (https://gtexportal.org/home/) gene expression data was performed using the GEPIA database. This database can also be used to analyze the differential gene expression observed between cancer and normal tissues [32]. Therefore, GEPIA (http://gepia.cancer-pku.cn/) was used to explore BUB1/3 mRNA expression in 33 types of cancer, including LUAD and its paired normal tissues.

The systematic TIMER database (http://timer.cistrome.org/) was used to determine the differential gene expression between tumors and adjacent normal tissues [33]. The microarray expression values are studied to arrive at the results. The results were visualized and the correlation of gene expression (with levels of immune invasion) were analyzed using this database.

### Differential expression of BUB gene family in LUAD at protein level

In addition to the mRNA expression analysis using the Oncomine, GEPIA, and TIMER databases, we also analyzed the data presented in the UALCAN and HPA databases to evaluate the protein expression levels of BUB3. However, we could not find data on the BUB1 expression levels in the HPA and UALCAN databases. Nevertheless, we analyzed the BUB3 protein expression levels observed in the LUAD and normal tissues and compared the results. The UALCAN database was analyzed to arrive at the results [34]. *P* values < 0.001 were considered to be statistically significant.

HPA is an online platform containing representative immunohistochemical images of protein expression data in different cancer types [35]. We obtained data of the representative immunohistochemical images of BUB3 protein expression in the case of LUAD and normal samples from the HPA.

### Construction of the BUB gene family-related gene network

GeneMANIA is a website that presents genomic and proteomic data that can be used to identify genes that share functions with a single query gene according to the interaction between genes [36]. We submitted the relevant data on BUB1/3 to GeneMANIA to evaluate the functional association network and the associated genes.

### GO enrichment and KEGG pathway enrichment analyses

WebGestalt is used to conduct functional enrichment analysis [37]. We used WebGestalt to analyze the GO functions and pathways associated with BUB1/3 and the 20 genes related to BUB1/3. The over-representation analysis (ORA) method was used for sample analysis. The enrichment of GO function was represented with respect to cellular component (CC), biological process (BP), and molecular function (MF). The KEGG analysis method was used for pathway analysis.

### Immunoinfiltration analysis of BUB gene family in LUAD

The infiltration analysis of BUB1/3 in LUAD was performed using the TIMER database [33]. Analysis of the generated scatter plot corresponding to BUB1/3 revealed the value of the fudge-corrected partial Spearman's Rho and the statistical significance. We observed that the expression levels of the positive purity expectation genes were significantly elevated in tumor cells. In contrast, the expression levels of the opposite expectation genes were elevated in the microenvironment.

### Clinicopathological analysis of the BUB gene family

The UALCAN database is used for the comprehensive analysis of TCGA gene expression data. This platform is a comprehensive interactive web resource that uses information on RNA sequences from TCGA database. Information on RNA sequences for 31 types of cancers is present in this database. Therefore, we used UALCAN to verify the accuracy of the obtained data on differential expression. The expression levels of BUB1/3 mRNA in LUAD and normal tissues were analyzed using TCGA–LUAD dataset (statistical significance: *p* <0.001).

The relationship between the clinicopathological parameters (such as individual cancer staging, lymph node metastasis status, and sex of the patients) and BUB1/3 mRNA or protein expression levels was also analyzed. The desired results can be readily obtained if the clinicopathological grouping option integrated into the database is selected. However, only the tumor group could be categorized into various clinicopathological groups. (statistical significance: *p* < 0.001).

### Receiver operating characteristic (ROC) curve analysis

The AUC (Area Under Curve) of the ROC curve was generated to evaluate the predictive value of the genes. AUC is between 0.5 and 1.0. The closer the AUC value is to 1.0, the better the diagnostic effect. The abscissa was the false positive rate (FPR), and the ordinate was the true positive rate (TPR) [38].

### Survival analysis

The GEPIA (http://gepia.cancerpku.cn/index.html) database was used for analyzing the prognostic value of the various BUB1/3 mRNA expression levels in LUAD. A total of 9,736 tumor samples and 8,587 normal samples were retrieved from the GTEx and TCGA databases. The median mRNA expression level was determined, and the patients with LUAD were divided into high and low expression groups (statistical significance: ***p*** < 0.05).

### Cell culture

Various cell lines were purchased to conduct the experiments. The human cell lines (H1299, PC9, A549, H460, BEAS-2B and the large-cell lung cancer cell line H460) were obtained from the Wuhan Boster Company, and the HBE cell line was bought from the Wuhan Procell Company. The cells were cultured using RPMI-1640 (Nanjing KeyGen Biotech Co., Ltd.) and 10% fetal bovine serum (Gibco; Thermo Fisher Scientific, Inc., USA). The H1299, A549, H460 and BEAS-2B cell lines were cultured, and Dulbecco's Modified Eagle Medium /high glucose supplemented containing 10% fetal bovine serum (HyClone; Cytiva) was used to culture the PC9 and HBE cell lines. The cells were cultured at a temperature of 37° C in a humidified incubator (Thermo Fisher Scientific, Inc.). The atmosphere of the container consisted of 21% O2, 5% CO2, and 74% N2.

### RNA extraction and RT-qPCR detection

The cells were treated under appropriate conditions to conduct the experiments. Trizol was used for harvesting the cells. The guidelines outlined by the manufacturer were followed to obtain the total RNA. Subsequently, the concentration of RNA was determined using the NanoDrop-2000 spectrophotometer (Thermo Fisher Scientific, Inc.). The stem-loop reverse transcriptase primer kit (Ribobio, Guangzhou, China) was used to realize the reverse transcription of the mRNA samples. The qRT–PCR technique was used for sample analysis, and the SYBR Prime Script Kit (Takara Bio Inc., Shiga, Japan) was used to conduct each experiment three times.

### Protein extraction and Western blot analysis

The total protein content of lysates was determined using the BCA protein assay kit (Pierce, Rockford, IL, USA). The proteins were isolated using the sodium dodecyl sulfate polyacrylamide gel. The isolated proteins were subsequently transferred to a polyvinylidene fluoride membrane. After protein transfer, the membrane was sealed with 5% skimmed milk powder. The sealed contents were incubated overnight with specific antibodies. An enhanced chemiluminescence reagent purchased from Thermo Fisher Scientific, Inc., USA, was used to visualize the results.

### Statistical analysis

R software (version V.3.5.1) was used for the statistical analyses and to generate the various plots. The expression of the BUB1/3 genes was studied using a t-test. The Wilcoxon signed- rank test, one-way ANOVA, and logistic regression were used to study the relationship between the clinicopathological features and BUB1/3 expression. The prognostic factors were analyzed using Cox regression analysis and Kaplan-Meier method.

### Availability of data and materials

The data that support the findings of this study were generated at TCGA, CPTAC, and HPA. Derived data supporting the findings of this study are available from the corresponding author on reasonable request.
